# Hippocampal mitophagy contributes to spatial memory via maintaining neurogenesis during the development of mice

**DOI:** 10.1111/cns.14800

**Published:** 2024-06-17

**Authors:** Le Xu, Saboor Saeed, Xinxu Ma, Xufeng Cen, Yifei Sun, Yanan Tian, Xuhong Zhang, Danhua Zhang, Anying Tang, Hetong Zhou, Jianbo Lai, Hongguang Xia, Shaohua Hu

**Affiliations:** ^1^ Department of Psychiatry, The First Affiliated Hospital Zhejiang University School of Medicine Hangzhou China; ^2^ Nanhu Brain‐computer Interface Institute Hangzhou China; ^3^ Research Center of Clinical Pharmacy of The First Affiliated Hospital & Liangzhu Laboratory Zhejiang University School of Medicine Hangzhou China; ^4^ Department of Biochemistry Zhejiang University School of Medicine Hangzhou China; ^5^ The Zhejiang Key Laboratory of Precision psychiatry Hangzhou China; ^6^ Brain Research Institute of Zhejiang University Hangzhou China; ^7^ Zhejiang Engineering Center for Mathematical Mental Health Hangzhou China; ^8^ MOE Frontier Science Center for Brain Science and Brain‐Machine Integration Zhejiang University Hangzhou China; ^9^ Department of Psychology and Behavioral Sciences Zhejiang University Hangzhou China

**Keywords:** development, mitophagy, neurogenesis, spatial memory

## Abstract

**Background:**

Impaired mitochondrial dynamics have been identified as a significant contributing factor to reduced neurogenesis under pathological conditions. However, the relationship among mitochondrial dynamics, neurogenesis, and spatial memory during normal development remains unclear. This study aims to elucidate the role of mitophagy in spatial memory mediated by neurogenesis during development.

**Methods:**

Adolescent and adult male mice were used to assess spatial memory performance. Immunofluorescence staining was employed to evaluate levels of neurogenesis, and mitochondrial dynamics were assessed through western blotting and transmission electron microscopy. Pharmacological interventions further validated the causal relationship among mitophagy, neurogenesis, and behavioral performance during development.

**Results:**

The study revealed differences in spatial memory between adolescent and adult mice. Diminished neurogenesis, accompanied by reduced mitophagy, was observed in the hippocampus of adult mice compared to adolescent subjects. Pharmacological induction of mitophagy in adult mice with UMI‐77 resulted in enhanced neurogenesis and prolonged spatial memory retention. Conversely, inhibition of mitophagy with Mdivi‐1 in adolescent mice led to reduced hippocampal neurogenesis and impaired spatial memory.

**Conclusion:**

The observed decline in spatial memory in adult mice is associated with decreased mitophagy, which affects neurogenesis in the dentate gyrus. This underscores the therapeutic potential of enhancing mitophagy to counteract age‐ or disease‐related cognitive decline.

## Graphical abstract

During the development of mouse, alterations in mitophagy within hippocampus induce the disparities in spatial memory duration between adolescent and adult mice via regulating neurogenesis.

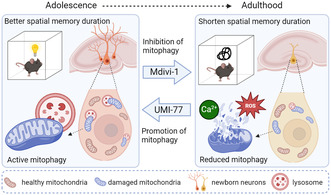

## 1 INTRODUCTION

The brain undergoes continuous changes from embryonic development, through adulthood, resulting in distinct behavioral characteristics at different life stages,^1^, ^2^, ^3^ significantly contributing to the age‐related manifestations of various neurological and psychiatric disorders.^4^, ^5^ Adolescence is a critical period for brain development, characterized by synaptic pruning and refinement, neurotransmitter regulation, and enhanced plasticity. These processes are essential in shaping behavior, cognition, and neurobiology,^5^ making the transition from adolescence to adulthood a vulnerable period for the onset of disorders such as schizophrenia, bipolar disorder, and major depressive disorder (MDD).^6^ Elucidating the functional changes in the brain during this developmental period may provide insights into the pathogenic mechanisms underlying age‐related neuropsychiatric disorders.

The hippocampal dentate gyrus (DG) serves as a crucial site of adult neurogenesis, where neural stem and progenitor cells differentiate into functional neurons. Even in adult animals, this process persists and is therefore known as adult neurogenesis.^7^ Despite the notable decrease in the number of newborn neurons during the transition from adolescence to adulthood,^8^, ^9^ evidence from animal research indicates that adult neurogenesis is believed to have a significant impact on the cognitive and emotional functions of individuals.^10^ Moreover, diseases like Alzheimer's disease (AD) have pathological mechanisms linked to deviations in the functionality or number of newborn neurons,^11^ others like MDD,^12^ and Parkinson's disease (PD).^13^ Understanding the factors contributing to the developmental decline in neurogenesis may enhance our understanding of disease pathogenesis and guide the exploration of novel therapeutic approaches.

Mitochondrial function and dynamics are crucial for regulating cell proliferation and differentiation.^14^ Mitochondrial metabolism and respiratory functions play significant roles in maintaining stemness and driving differentiation within stem cells.^15^, ^16^ Consequently, the regulation of mitochondria also impacts hippocampal neurogenesis.^17^, ^18^ In animal models of aging^19^ and various diseases, impaired mitochondrial function plays a crucial role in the decline in neurogenesis. This is evident in conditions such as AD,^20^ dominant optic atrophy,^21^ and PD.^22^ Mitochondrial dynamics, including fusion, fission, and mitophagy, are essential for maintaining overall mitochondrial health, thus playing a significant role in the regulation of neurogenesis. For example, in mice with a heterozygous knockout of the mitochondrial fusion protein Opa‐1, a dysfunction in hippocampal neurogenesis occurs, leading to impaired spatial memory^21^; ganglioside GD3 regulates adult hippocampal neurogenesis through the turnover modulation of the mitochondrial fission protein Drp1^23^; and mice with a knockout of the ubiquitin‐dependent mitophagy protein PTEN‐induced kinase 1 (PINK1) exhibit abnormalities in hippocampal metabolism and neurogenesis.^24^ The exact effect of mitochondrial dynamics on changes in neurogenesis throughout the transition from adolescence to adulthood is not yet fully understood.

In summary, the developmental trajectory of the brain holds crucial significance for individual behavior, emotions, and the pathology of neurodegenerative diseases and psychiatric disorders, with potential involvement of hippocampal neurogenesis. In neurological and psychiatric disorders, abnormalities in mitochondrial dysfunction and dynamics emerge as pivotal factors contributing to impaired neurogenesis. We hypothesize that alterations in mitochondrial dynamics within the hippocampus during development may underlie the gradual reduction in newborn neurons, potentially mediating behavioral differences observed between adolescence and adulthood. We assessed these changes in adolescent and adult mice, and shed light on the relationship among mitochondrial dynamics, neurogenesis, and behavioral performance through the administration of compounds targeting mitophagy.

Our findings indicate that the downregulation of mitophagy during the transition from adolescence to adulthood may be a primary cause of decreased neurogenesis, consequently resulting in shortening spatial memory duration in adult mice compared to adolescent mice.

## 2 MATERIALS AND METHODS

### 2.1 Animals

All animal experiments were conducted with approval from the Animal Experimentation Ethics Committee of the First Affiliated Hospital, Zhejiang University School of Medicine. C57/BL6J mice were purchased from Gempharmatech Co., Ltd. Mice were housed in groups of 3–5 per cage with a 12‐hour light–dark cycle and ad libitum access to water and food. The housing environment was maintained at a temperature of 20–22°C and a humidity of 45%–55%.

### 2.2 Behavioral tests

All behavioral experiments were conducted during the dark phase of the mice, and began for adolescent mice between postnatal days 40 and 45 (P40‐45) and for adult mice between P63‐77. Three days prior to the behavioral tests, each mouse was habituated to the behavioral room, and a single experimenter handled subjects for 5 min per day to mitigate stress. On the day of the behavioral experiment, mice were placed in the experimental room 1 h in advance. Following completion of each behavioral test, the apparatus was wiped with 75% alcohol to prevent odor interference with behavioral outcomes. The analysis was carried out by investigators unaware of the grouping and treatment of the mice.

#### 2.2.1 Open field test (OFT)

The OFTs were conducted to evaluate both locomotor activity and anxiety‐related behavior. The open field apparatus, constructed from white Plexiglas plates, measured 40 cm in length, width, and height. Mice were introduced into the center of the open field and given a 10‐min period for free exploration. Locomotor activity was recorded by a camera situated above the open field. The total distance traveled and the duration spent in the central zone during this session were subsequently analyzed and depicted as movement trajectories using ALS Vision software (AniLab Scientific Instruments Co., Ltd., China).

#### 2.2.2 Novel location recognition (NLR)/Novel object recognition (NOR)

The NLR and NOR tests were performed to test recognition memory in mice. In the NLR test, the device includes a small open field box (25 cm L × 25 cm W × 25 cm H, one of whose walls is specially marked) and two identical objects. Mice were acclimated to the open field box for 10 min on the 1st day. During training, 24 h after the acclimatization, mice were allowed to explore two identical objects for 10 min. Investigation time for each object was measured. One hour after training for test, one of the objects was picked up and placed diagonally opposite the other and mice were allowed again to explore two objects for 5 min. The investigation time for each object was measured again. Object exploration time was measured for each case in which a mouse's nose touched the object or was oriented toward the object and came within 2 cm of it. The NLR discrimination index, reflecting spatial recognition memory ability, was defined as (novel location investigation time − familiar location investigation time)/(novel location investigation time + familiar location investigation). The device for NOR test includes an open field box (25 cm L × 25 cm W × 25 cm H) and two objects of different shapes but of the same material. The acclimating and training phases are the same as NLR test in the first 4 days. On the 5th day (24 h after the training phases), one of the objects was replaced with a new object, mice were allowed to explore two different objects for 5 min. The data are recorded in the same way as NLR test. The NOR discrimination index, reflecting object recognition memory, was defined as (novel object investigation time − familiar object investigation time)/(novel object investigation time + familiar object investigation time).

#### 2.2.3 Y‐maze

The Y‐maze tests were performed to assess short‐term spatial working memory by spontaneous alternation. The device is a three‐arm maze (40 cm L and 10 cm W with 25 cm H) in which the angle between each of the two adjacent arms is 120°. Mice were placed at the junction of the three arms and allowed to explore freely for 8 min. The total entries and sequence of arms were recorded. The percent alternations, reflecting spatial working memory ability, were defined as the proportion of arm choices that differed from the last two choices.

#### 2.2.4 Nonassociative place recognition (NAPR)

The NAPR tests serve to evaluate spatial memory in mice by observing changes in their locomotor activity when introduced twice to the same area. Initially, on the 1st day, mice were allowed 10 min of free exploration in the open field, during which their total distance was recorded (following the methodology detailed in Section 2.2.1 for OFT). This phase marked the stage of spatial memory acquisition. Following intervals of 1 day/28 days/35 days, mice were reintroduced to the same open field for another 10‐minute session of free exploration, during which their spontaneous activity was once again recorded, representing the recall phase. If the mice retained spatial memory from the initial phase during the recall phase, their exploratory behavior within the open field diminished, as indicated by a decrease in total distance. To account for potential effects of drug interventions on activity levels, 1 day after the second phase, mice were placed in an open field with a different shape but equal size (circular bottom; 22.57 cm *R* ^2^ × 40 cm H) for the evaluation of spontaneous activity.

### 2.3 Drug

The experiment involved the administration of UMI‐77 (HY‐18628, MedChemExpress) to mice via intraperitoneal injection at a dosage of 5 mg/kg per day for 14 days or 49 days (in the NAPR trial). UMI‐77 was dissolved in saline containing 2% DMSO (HY‐Y0320, MedChemExpress) and 30% PEG 400 (P8530, Solarbio). Mdivi‐1 (HY‐15886, MCE) was administered through two routes: intracerebral injection (200 μM, 0.5 μL, unilateral or bilateral) and intraperitoneal injection (20 mg/kg). In the intracerebral administration, bilateral cannulas (Yuyan Instruments) were implanted into the hippocampal DG (AP: −2.0, ML: ±1.0, DV: 2.0). Three days post‐surgery, mice were given Mdivi‐1 at a rate of 0.1 μL/min for 7 consecutive days, with the drug dissolved in 0.3% DMSO and 10% PEG‐400. In the intraperitoneal administration, the drug was administered for 7 weeks, dissolved in saline containing 2% DMSO and 30% PEG‐400.

### 2.4 Immunofluorescence

Prior to transcardial perfusion with saline followed by 4% paraformaldehyde (PFA), the mice were anesthetized using pentobarbital sodium (80 mg/kg). Post decapitation, the brains were fixed in 4% PFA for 24 h and subsequently dehydrated for 2 days in a phosphate‐buffered saline (PBS) solution containing 30% sucrose. The brains were then sliced into 40 μm sections using a Cryostat (NX50, Epredia). Sections were blocked via immersing into a blocking solution (0.1% TritonX‐100 and 1% BSA in PBS) at room temperature for 1 h, followed by an overnight incubation at 4°C with primary antibodies diluted in the same solution. After washing with PBS, the sections underwent a 1‐h incubation at room temperature (RT) with secondary antibodies (1:500, 8889S/8890S, Cell Signaling Technology) diluted in PBS. Subsequent steps involved PBS washes and covering the sections with an anti‐fade mounting medium (S2110, Solarbio). We used the following primary antibodies for immunofluorescence: anti‐DCX (1:1000, 4604, Cell Signaling Technology), anti‐BrdU (1:500, 5292, Cell Signaling Technology), anti‐NeuN (1:500, 24307, Cell Signaling Technology), anti‐GFAP (1:500, 16825‐1‐AP, Proteintech), and anti‐Iba1(1:500, 17198, Cell Signaling Technology). To evaluate neurogenesis levels in mice using BrdU staining, additional steps were introduced: mice received an intraperitoneal injection of BrdU (S7918, Selleck) at 100 mg/kg 24 h before tissue collection. Brain sections were subjected to a 15‐min incubation in 2 M HCL at 37°C, followed by three 5‐minute washes in borate buffer (pH = 8.4) and subsequent washes in PBS. The sections then underwent the same blocking and primary/secondary antibody incubation steps as previously described. The outcome was observed using a Leica DM4B microscope. The Olympus VS200 Slide Scanner was used to scan the whole brain section to view the staining outcomes. ImageJ software (version 1.52a; National Institutes of Health) was used to quantify the fluorescent signal indicative of positive immunostaining.

### 2.5 Western blotting

The mice were deeply anesthetized using pentobarbital (80 mg/kg, i.p.) and subsequently decapitated. Following this, hippocampal tissues were directly lysed in sodium dodecyl sulfate sample buffer and incubated at 95°C for 5 min before loading onto a 10% sodium dodecyl sulfate–polyacrylamide gel. Protein concentrations were determined prior to gel loading, with 40 μg of protein per sample loaded onto each track. Electrophoresis was conducted to separate the proteins, followed by their transfer to a nitrocellulose membrane (BioRad, Hercules, CA, United States). The membrane underwent blocking in 5% BSA TBST at RT and was probed with primary antibodies overnight at 4°C. Subsequently, the membrane was incubated with Alexa Fluor 800‐conjugated antibody (1:5000) for 60 min. Specific band detection and quantification were performed using ImageJ software. The primary antibodies included anti‐Parkin (1:1000, A0968, ABclonal), anti‐PINK1 (1:1000, A11435, ABclonal), anti‐LC3B(1:1000, A19665, ABclonal), anti‐Drp1(1:1000, HA500487, HUABIO), anti‐Opa1 (1:1000, 80,471, Cell Signaling Technology), anti‐Mfn1 (1:1000, A21293, ABclonal), anti‐p62 (1:1000, R1309‐8, HUABIO), anti‐FUNDC1 (1:1000, A16318, ABclonal), anti‐NIX/BNIP3L (1:1000, 12,396, Cell Signaling Technology), and anti‐BNIP3 (1:1000, A5683, ABclonal). All samples underwent analysis at least in triplicate, with β‐actin (AC026, ABclonal, China; 1:5000) used as the internal protein control.

### 2.6 Transmission electron microscopy (TEM)

#### 2.6.1 Sample preparation

Mice were anesthetized and subsequently perfused with a solution containing 2.5% glutaraldehyde and 2% paraformaldehyde. The brains were then removed, and specific target tissues were isolated and rinsed in 0.1 M phosphate buffer (PB). These tissues underwent a post‐fixation process using 2.5% glutaraldehyde for 2 h at RT. Following two washes with 0.1 M PB, the samples were finally fixed using 1% osmium tetraoxide for 5 min at RT. Subsequently, the samples underwent a dehydration process in a sequential manner: 50%, 70%, and 90% ethanol solutions, then a mixture of 90% alcohol and 90% acetone for 10 min each, followed by graded acetone up to 100% for another 10 min. General staining was performed using Epon 812 in 100% acetone for 2 h. Following this, tissues were infiltrated and embedded in Epon 812, and the embedding was polymerized at 60°C for 24 h. Sections (400 nm) of the hippocampus were then cut using a diamond knife, post‐stained with uranyl acetate and lead citrate, and observed using a Tecnai G2 Spirit Transmission Electron Microscope (Thermo FEI) operating at 120 kV.

#### 2.6.2 Quantification of TEM

Subcellular structures bound by a double limiting membrane and containing discernible mitochondria, such as presence of double membranes and cristae, were identified as putative mitophagosome‐like structures.

### 2.7 Statistical analysis

GraphPad Prism version 9 (GraphPad Software, San Diego, CA, United States) was used to conduct statistical analyses. All data were assessed for distribution using the Shapiro–Wilk normality test. When the data followed a normal distribution, an unpaired *t*‐test was employed to compare the means of two independent sample groups, while a paired *t*‐test was utilized to compare the means of paired samples. Otherwise, non‐parametric tests were employed, with the Mann–Whitney test used for two independent sample groups and the Wilcoxon test used for paired samples. Two‐way ANOVA followed by Bonferroni's post hoc test when appropriate was used to analyze the spontaneous activity of two groups of mice in the NAPR tests. Data were presented as mean ± SEM. *p* < 0.05 was considered statistically significant for all results.

## 3 RESULTS

### 3.1 Adolescent mice performed better on spatial recognition memory

**FIGURE 1:**
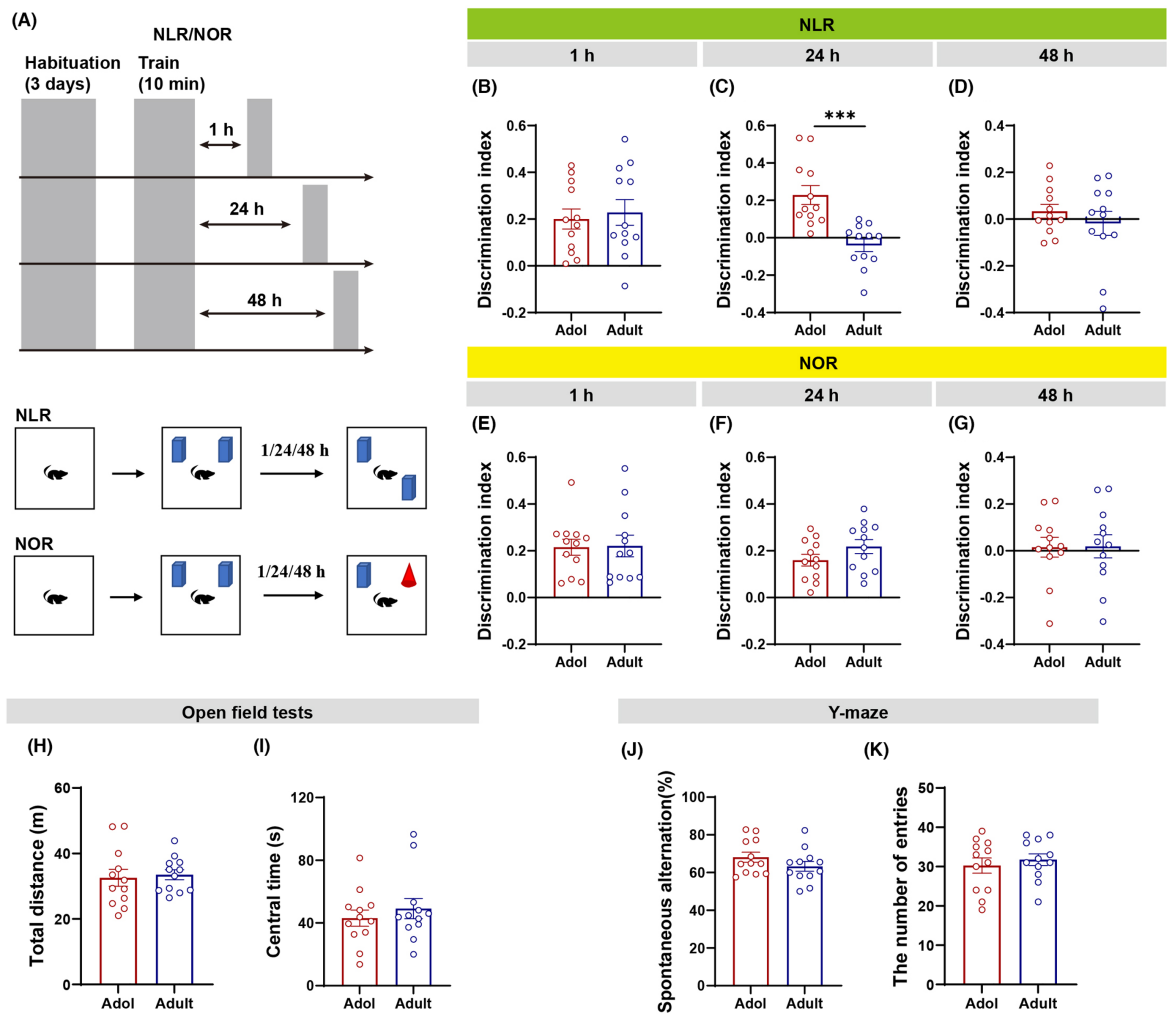
Cognitive‐behavioral performance in male C57 mice during adolescence and adulthood. (A) Experimental diagram of NLR and NOR. (B–D) Adult mice showed decreased discrimination index at 24 h after training in NLR compared to adolescent mice (*n* = 12 mice/group; unpaired *t*‐test, *t*_(22)_ = 4.446, ***p = 0.0002), but no significant difference was observed at 1 h and 48 h after training (*n* = 12 mice/group). (E–G) In the three periods after training, no significant differences in discrimination index in NOL were observed between adolescent and adult mice (*n* = 12 mice/group). (H, I) In the OFT, there was no statistical difference in the total distance and central time before and after adulthood of mice (*n* = 12 mice/group). (J, K) Adolescent and adult mice showed comparable performance in the spontaneous alternation and total entries of Y‐maze (*n* = 12 mice/group).

We tested adolescent and adult male mice for cognitive and behavioral differences using the Y‐maze, NLR, NOR, and OFT. The NLR and NOR assessments were structured into three phases: habituation, training, and testing. Notably, the interval between the training and testing phases varied across different studies. To address this variability, we implemented protocols with diverse time intervals, aiming to precisely delineate subtle cognitive differences during the transition from adolescence to adulthood in male mice. For the NLR and NOR evaluations (Figure 1A), mice were acclimated to a specially designed open field for 3 days, engaging in 10‐min sessions daily. On the subsequent day, they were introduced to two identical objects during the training phase. Following this, the subjects were tested at intervals of 1, 24, or 48 h post‐training, herein referred to as 1/24/48 h‐NLR/NOR. When comparing adolescent to adult mice, we exclusively observed a significant decline in performance in adult mice during the 24‐h NLR test (Figure 1C). Conversely, no significant differences were detected in the discrimination indices between the groups in the 1‐h and 48‐h NLR assessments (Figure 1B,D). Likewise, both groups demonstrated equivalent recognition memory in the NOR assessment at all post‐training intervals (Figure 1E–G). Furthermore, the OFT findings indicated that there were no significant differences in locomotor activity between the adult and adolescent mice (Figure 1H). Both groups visited the central area for similar amounts of time (Figure 1I). Furthermore, the Y‐maze test, which is employed to evaluate working memory, did not identify any statistically significant differences between the two groups in terms of spontaneous alternation or total entries (Figure 1J,K).

The results indicate that adolescent mice might exhibit better spatial recognition memory performance compared to adults. However, no statistically significant differences were detected in object recognition memory, working memory, locomotor activity, or anxiety‐like behavior among the different groups.

### 3.2 Prolonged spatial memory in the adolescence of mice

**FIGURE 2:**
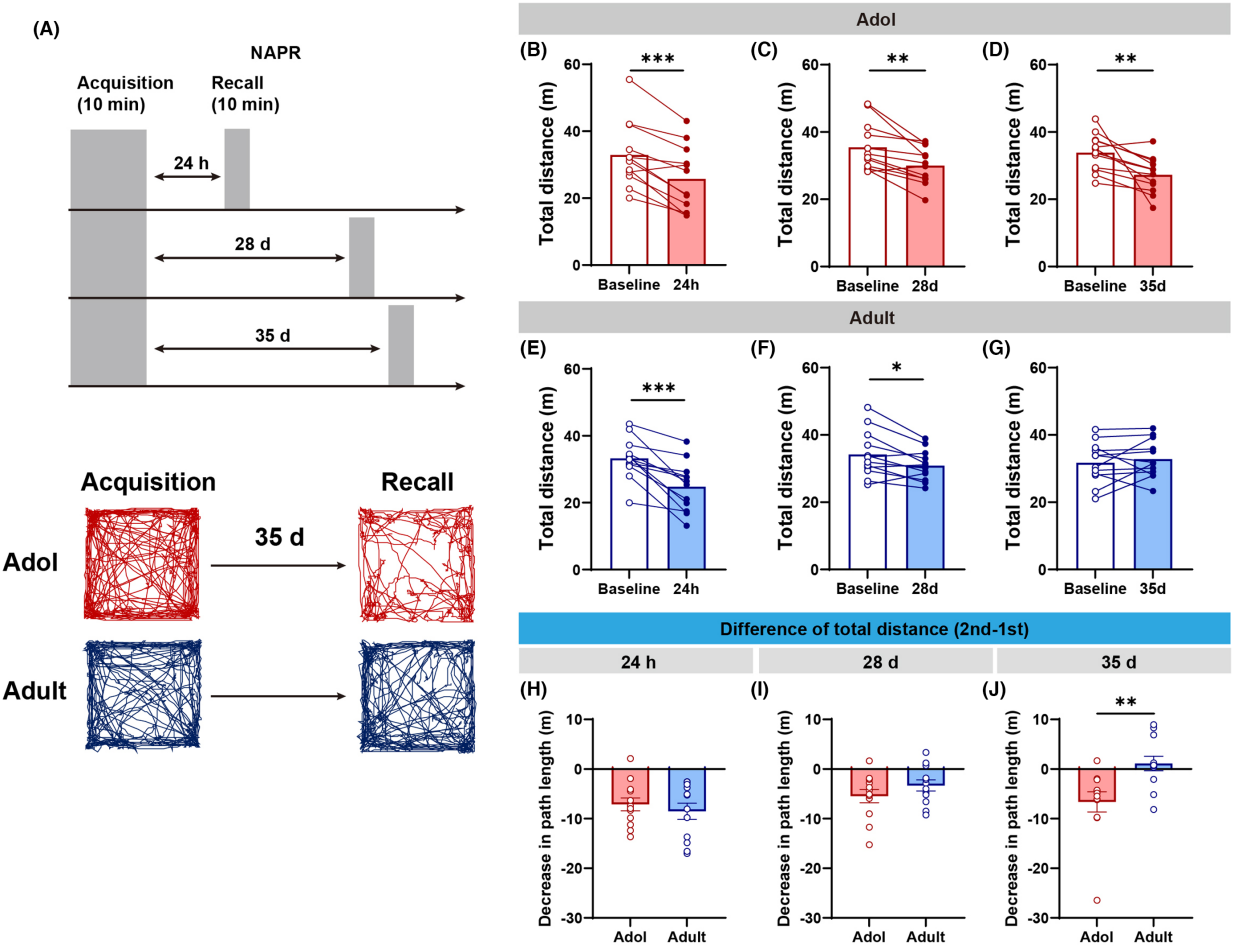
Differences in spatial memory between adolescence and adulthood of male C57 mice. (A) Experimental diagram (top) and example exploration traces of NAPR (bottom); male C57 mice were explored in open field twice at different intervals (24 h, 28 days, and 35 days); “acquisition” means visit 1, “recall” means visit 2; the motion trajectories of adolescent and adult mice were demonstrated in 35‐day interval NAPR. (B–D) In the three intervals of the NAPR, adolescent mice showed a significant decrease in the exploration when visiting open field again (*n* = 12 mice/group; for 24 h, paired *t*‐test, *t*_(11)_ = 5.438, ***p = 0.0002; for 28 days, paired *t*‐test, *t*_(11)_ = 4.087, **p = 0.0018; for 35 days, paired *t*‐test, *t*_(11)_ = 3.273, **p = 0.0074). (E–G) In NAPR, adult mice showed decreased locomotor activation in recall sessions at intervals of 24 h and 28 days, but comparable performance in two visits at intervals of 35 days (*n* = 12 mice/group; for 24 h, paired *t*‐test, *t*_(11)_ = 5.224, ***p = 0.0003; for 28 days, paired *t*‐test, *t*_(11)_ = 2.973, *p = 0.0127). (H–J) The average change (Δ) of total distance in two visits showed significant differences only before and after 35 days, but not seen at intervals of 24 h and 28 days (*n* = 12 mice/group; for 35 days, Mann–Whitney test, **p = 0.0036).

To further validate the difference in spatial memory between adolescence and adulthood of male C57 mice, we utilized the NAPR paradigm. In this task, as previously reported,^25^, ^26^ when mice are introduced to the same environment twice, it is expected that exploration of this familiar environment will significantly diminish due to acquired memory. As illustrated in Figure 2A, “Acquisition” represents the initial exposure to a novel open field, and “Recall” refers to subsequent re‐entries into the same field, spaced by intervals designed to test the longevity of spatial memory (Figure 2A).

Recent studies have demonstrated that adolescent mice showed a significant decrease in exploration levels upon re‐entering the same arena at 24 h, 28, and 35 days (Figure 2B–D). In contrast, adult mice exhibited reduced exploration at intervals of 24 h and 28 days (Figure 2E,F), but returned to initial exploration levels after a 35‐day interval (Figure 2G). These findings suggest that the spatial memory retention in adolescent mice extended beyond 35 days from their initial exposure, whereas adult mice retained the spatial memory of the arena for less than 35 days.

Moreover, comparisons were conducted between the change of total distance covered by adult and adolescent mice during successive entry periods (decrease in path length = visit 2 – visit 1), showing marked disparity at interval of 35 days (Figure 2J), but not 24 h and 28 days (Figure 2H,I). The results demonstrated that adolescent mice outperformed adult mice in terms of spatial memory duration.

### 3.3 Neurogenesis and mitophagy decline in the hippocampus of adult mice

**FIGURE 3:**
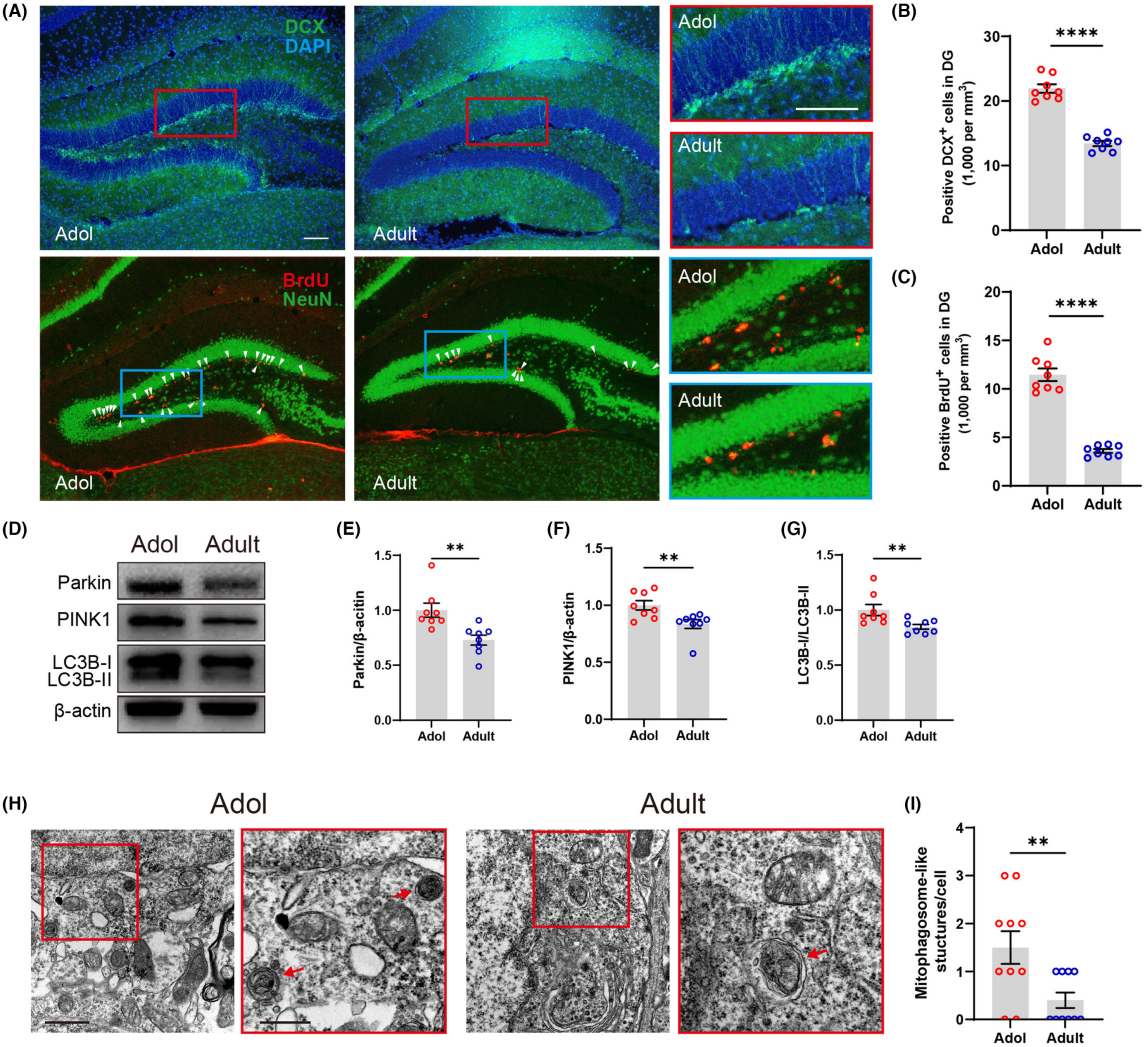
Changes in levels of neurogenesis and mitophagy from adolescence to adulthood in male C57 mice. (A) Representative images of DCX, BrdU and NeuN expression in hippocampal DG of adolescent mice and adult mice; scale bar (both in rimless and red/blue edge images), 100 μm. (B, C) The quantitative results of the DCX and BrdU staining showed a decrease in the number of young neurons and stem cell proliferation of hippocampus from adolescence to adulthood of mice (*n* = 8 mice/group; for DCX, unpaired *t*‐test, *t*_(14)_ = 11.06, ****p < 0.0001; for BrdU, unpaired *t*‐test, *t*_(14)_ = 11.57, ****p < 0.0001). (D) Exemplary Western blots depicting mitophagy‐associated proteins in the hippocampus. (E–G) Quantitative analysis revealed notable reductions in Parkin and PINK1 expression levels, along with a significant decrease observed in the expression ratio of LC3B‐II/LC3B‐I within the hippocampus of adult mice compared to adolescent mice (*n* = 8 mice/group; for Parkin, Mann–Whitney test, **p = 0.0011; for PINK1, Mann–Whitney test, **p = 0.007; for LC3B, Mann–Whitney test, **p = 0.0047). (H) Representative electron micrographs of mitophagosome‐like structures; red arrows indicate mitophagosome‐like structures; scale bar in rimless images, 1 μm; scale bar in red edge images, 500 nm. (I) Ultrastructural features assessed by electron microscopy showed decreased number of mitophagosome‐like structures per neuron within the hippocampus of adult mice compared to adolescent mice (*n* = 10 neurons from 3 mice/group; Mann–Whitney test, *p = 0.0234).

To investigate why spatial memory differs between adolescent and adult mice, we focused on hippocampal neurogenesis, a key factor in spatial memory.^21^, ^23^, ^27^ We used 5‐bromodeoxyuridinc (BrdU) to tag the cells added to the DG within 24 h, and doublecortin (DCX) served as a marker for young neurons aged between 3 days and 3 weeks. Our results, shown in Figure 3A–C, indicate that adult mice had significantly fewer newborn neurons than adolescent mice.

Given that mitochondria dynamics may be associated with adult neurogenesis in animal disease models,^21^, ^22^ we assessed the level of mitochondria dynamics in hippocampus during adolescence and adulthood of male C57 mice. Western blot analysis showed decreased levels of PTEN‐induced putative kinase1 (PINK1) and Parkin in the hippocampus during adulthood compared to adolescence in mice (Figure 3D–F). Additionally, we observed a decrease in the autophagic protein ratio LC3B‐II/LC3B‐I (Figure 3G), and P62 (an adaptor protein in autophagy) when mice entered adulthood (Figure S1E). However, the levels of receptor‐dependent mitophagy proteins, such as NIX and BNIP3, showed no significant difference between adolescence and adulthood of male C57 mice (Figure S1A,F–H). TEM further showed a reduction in mitophagosome‐like structures in adult mice (Figure 3H,I). These results indicate a significant reduction in the ubiquitin‐dependent mitophagy in adult mice. Moreover, a decline in the mitochondrial fusion proteins OPA1 and Mfn1 was observed in adults, while levels of the fission protein Drp1 remained constant (Figure S1B–D). These findings demonstrate significant variations in mitochondrial dynamics between adolescent and adult mice, presumably contributing to the decreased neurogenesis and impairment in spatial memory as mice age.

### 3.4 Promotion of mitophagy facilitates hippocampal neurogenesis in adult mice

**FIGURE 4:**
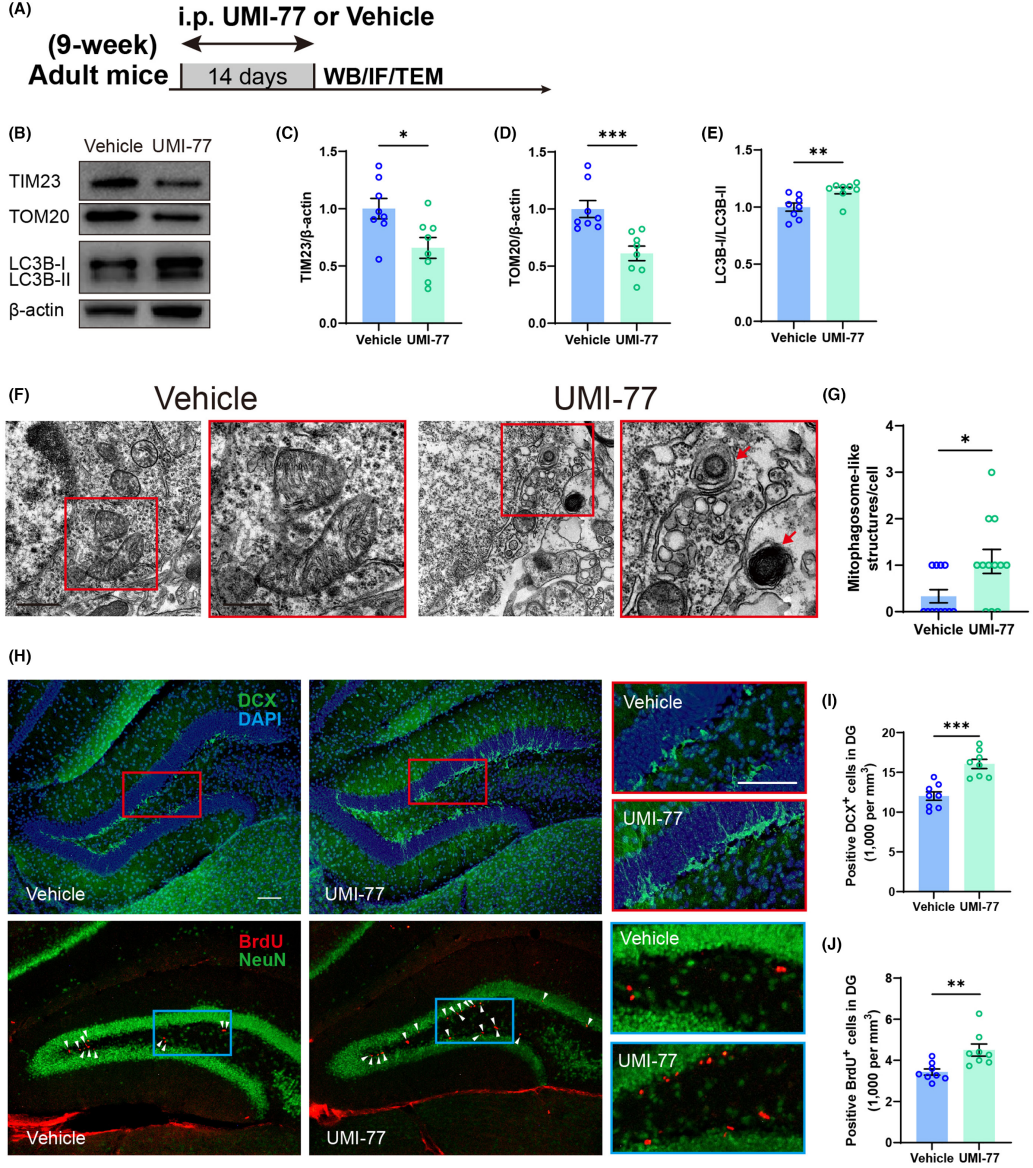
Effects of UMI‐77 on mitophagy and newborn neurons in adult male mice. (A) Experimental diagram of drug use. (B) Representative western blots of mitochondria/mitophagy‐related proteins in the hippocampus after chronic treatment of UMI‐77 or vehicle. (C–E) Quantitative analysis revealed reduced expression levels of TIM23 (without statistical significance) and significantly lowered levels of TOM20, accompanied by a noticeable elevation in the expression ratio of LC3B‐II/LC3B‐I within the hippocampus of adult mice following chronic treatment with either UMI‐77 or the vehicle (*n* = 8 mice/group; for TIM23, unpaired *t*‐test, *t*_(14)_ = 2.679, *p = 0.018; for TOM20, Mann–Whitney test, **p = 0.0002; for LC3B, Mann–Whitney test, **p = 0.0047). (F) Representative electron micrographs of mitophagosome‐like structures; red arrows indicate mitophagosome‐like structures; scale bar in rimless image, 1 μm; scale bar in red edge image, 500 nm. (G) Quantitative analysis from electron microscopy demonstrated a significant increase in the mitophagosome‐like structures observed in hippocampus of adult mice following chronic systemic administration of UMI‐77 (*n* = 12 neurons from 3 mice/group; Mann–Whitney test, *p = 0.0366). (H) Representative images of DCX, BrdU, and NeuN expression in hippocampal DG of adult mice after treatment of UMI‐77 or vehicle; scale bar (both in rimless and red/blue edge images), 100 μm. (I, J) Quantification from DCX and BrdU staining revealed increased number of newborn neurons in the hippocampal DG after treatment of UMI‐77 (*n* = 8 mice/group; for DCX, unpaired *t*‐test, *t*_(14)_ = 5.087, ***p = 0.0002; for BrdU, unpaired *t*‐test, *t*_(14)_ = 3.24, **p = 0.0059).

Preliminary observations suggest a potential regulatory interaction between mitophagy and neurogenesis within the hippocampal region. This hypothesis was investigated through the administered UMI‐77, a drug supposed to target the MCL‐1 receptor and facilitate mitophagy,^28^ to adult C57 mice via intraperitoneal injection (5 mg/kg) for 2 weeks. Mitochondrial marker proteins in the hippocampus were subsequently evaluated to confirm the induction of mitophagy (Figure 4A,B). The results revealed significant reductions in the TIM23 and TOM20, proteins of the inner and outer mitochondrial membranes, respectively, and a significant increase in the LC3B‐II/LC3B‐1 ratio, indicative of autophagic activity (Figure 4C–E). TEM confirmed a marked increase in mitophagosome‐like structures in the hippocampal neurons of the mice treated with UMI‐77 (Figure 4F,G), indicating the clearance of damaged mitochondria. Additionally, an increase in neurogenesis was observed as evidenced by elevated DCX and BrdU staining in the hippocampus following UMI‐77 treatment (Figure 4H–J), supporting the notion that mitophagy may regulate neurogenesis.

### 3.5 Promotion of mitophagy enhanced spatial memory in adult mice

**FIGURE 5:**
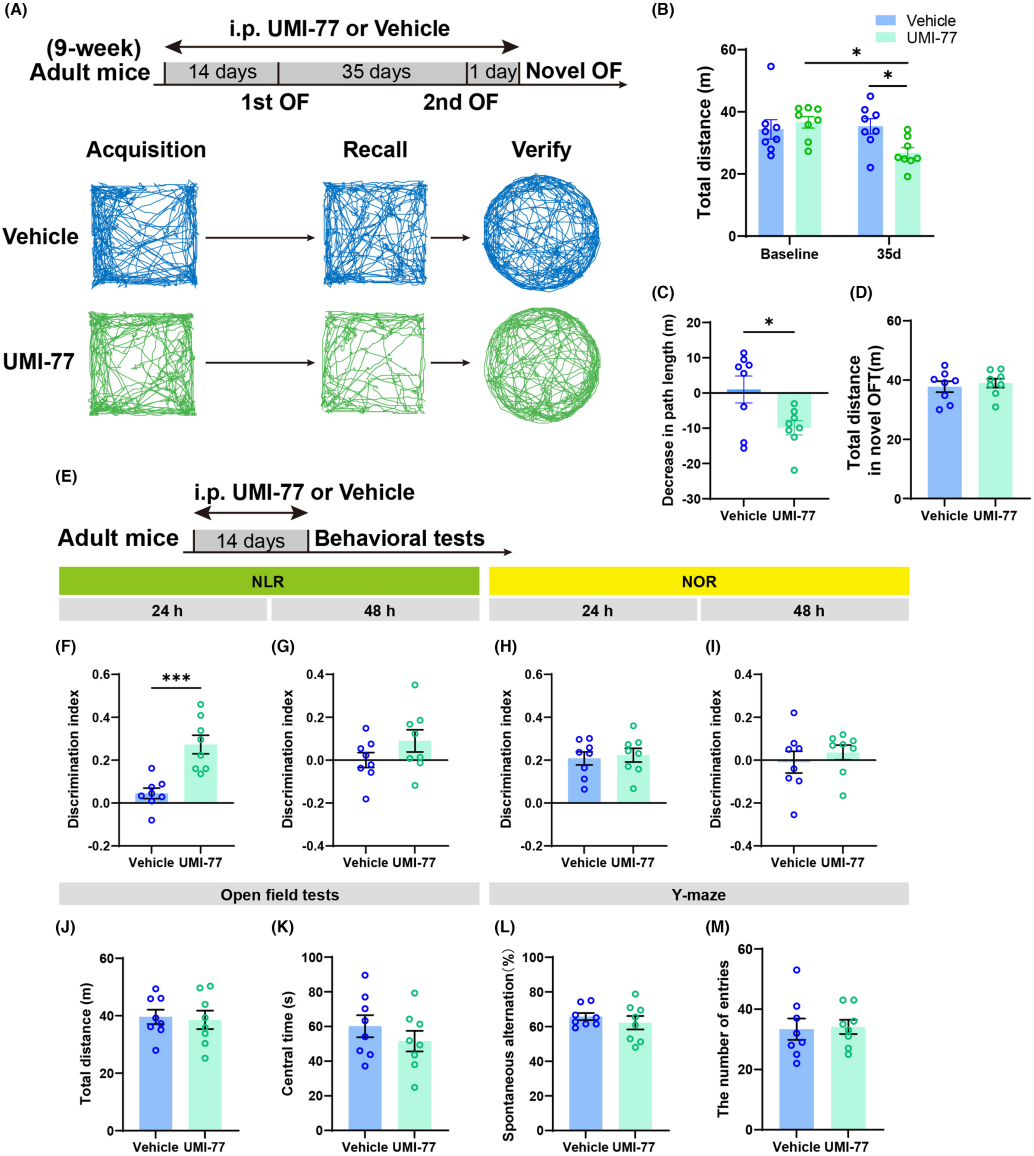
Changes in spatial memory and cognitive‐behavioral performance in adult male mice after chronic treatment of UMI‐77. (A) Experimental diagram (top) and example exploration traces of NAPR (bottom); mice were intraperitoneally injected with UMI‐77 for 2 weeks, after which the same open field was explored on day 1 and day 35, and another novel open field was explored on day 36. The drug continues to be maintained during the behavioral period. “Acquisition” means visit 1 in open field, “recall” means visit 2 in the familiar open field, and “verify” means visit 3 in novel open field. (B) In the 35‐day interval NAPR experiment, the UMI‐77‐treated mice exhibited a significant decrease in locomotor activity upon their second entry into the open field compared to their initial entry, as well as when compared to the second entry of the vehicle‐treated mice (*n* = 8 mice/group; two‐way ANOVA followed by Bonferroni's post hoc test, interaction of two factors: *F*_(1,28)_ = 5.292, *p* = 0.0291; for two groups after treatment of UMI‐77, baseline vs. 35 days, *p = 0.0126; for two groups tested after 35 days, vehicle vs. UMI‐77, *p = 0.0312). (C) The average change (Δ) of total distance between the first two visits showed significant differences between treatments of UMI‐77 and vehicle (*n* = 8 mice/group; unpaired *t*‐test, *t*_(14)_ = 2.514, *p = 0.0248). (D) In the novel open field, the third visit, there was no statistically significant difference in the total distance traveled, regardless of whether subjects received treatment with UMI‐77 or not (*n* = 8 mice/group). (E) Experimental diagram of cognitive‐related behavioral tests when using UMI‐77. (F, G) After administration of UMI‐77 to adult mice, a significant improvement in discrimination index of NLR was observed in contrast to the vehicle group at 24 h after training, but no significance at 48 h after training (*n* = 8 mice/group; for 24 h, unpaired *t*‐test, *t*_(14)_ = 4.574, *p = 0.0004). (H, I) In the NOR, there were no significant differences observed in the discrimination index between the UMI‐77 group and the vehicle group, both at 24 h and 48 h post‐training (*n* = 8 mice/group). (J, K) There was no statistical difference in the total distance and central time of OFT between adult mice administered with UMI‐77 and vehicle (*n* = 8 mice/group). (L, M) The UMI‐77 group showed comparable performance in the spontaneous alternation and total entries of Y‐maze to the vehicle group (*n* = 8 mice/group).

To evaluate the effect of mitophagy on spatial memory, behavioral tests were conducted following the administration of UMI‐77 to adult mice. Mice received systematic injections of UMI‐77 over a 2‐week period, followed by a 35‐day interval NAPR test while maintaining the drug regimen (Figure 5A). It was observed that locomotor activity in the UMI‐77‐treated mice significantly decreased upon their second entry into the same arena compared to the vehicle group (Figure 5B). Additionally, the difference in the total distance of two entries between UMI‐77 and vehicle group was statistically significant (Figure 5C). However, no difference in total distance covered was noted in a new open field test between the two groups (Figure 5D), suggesting that the reduced distance in the familiar open field by the UMI‐77 group was not attributable to decreased mobility caused by the drug, but rather to diminished exploratory behavior due to retained spatial memory.

In line with these observations, in the NLR, mice subjected to the 2‐week UMI‐77 intervention displayed a significantly higher discrimination index for the novel location 24 h after training compared to the controls (Figure 5E). However, no significant differences were found in recognition indices from the 48 h NLR and both the 24 and 48 h NOR (Figure 5G–I). Additionally, UMI‐77 did not alter the total movement distance in the open field test (Figure 5J), time spent in the central area (Figure 5K), spontaneous alternation (Figure 5L), and total entries in the Y‐maze (Figure 5M).

These findings indicate that UMI‐77 administration in adult mice prolongs the duration of spatial memory without affecting object recognition memory, working memory, or anxiety‐like behaviors. This supports the hypothesis that enhanced mitophagy may promote neurogenesis and improve spatial memory in adult mice.

### 3.6 Inhibition of mitophagy diminished neurogenesis and shortened spatial memory in adolescent mice

**FIGURE 6:**
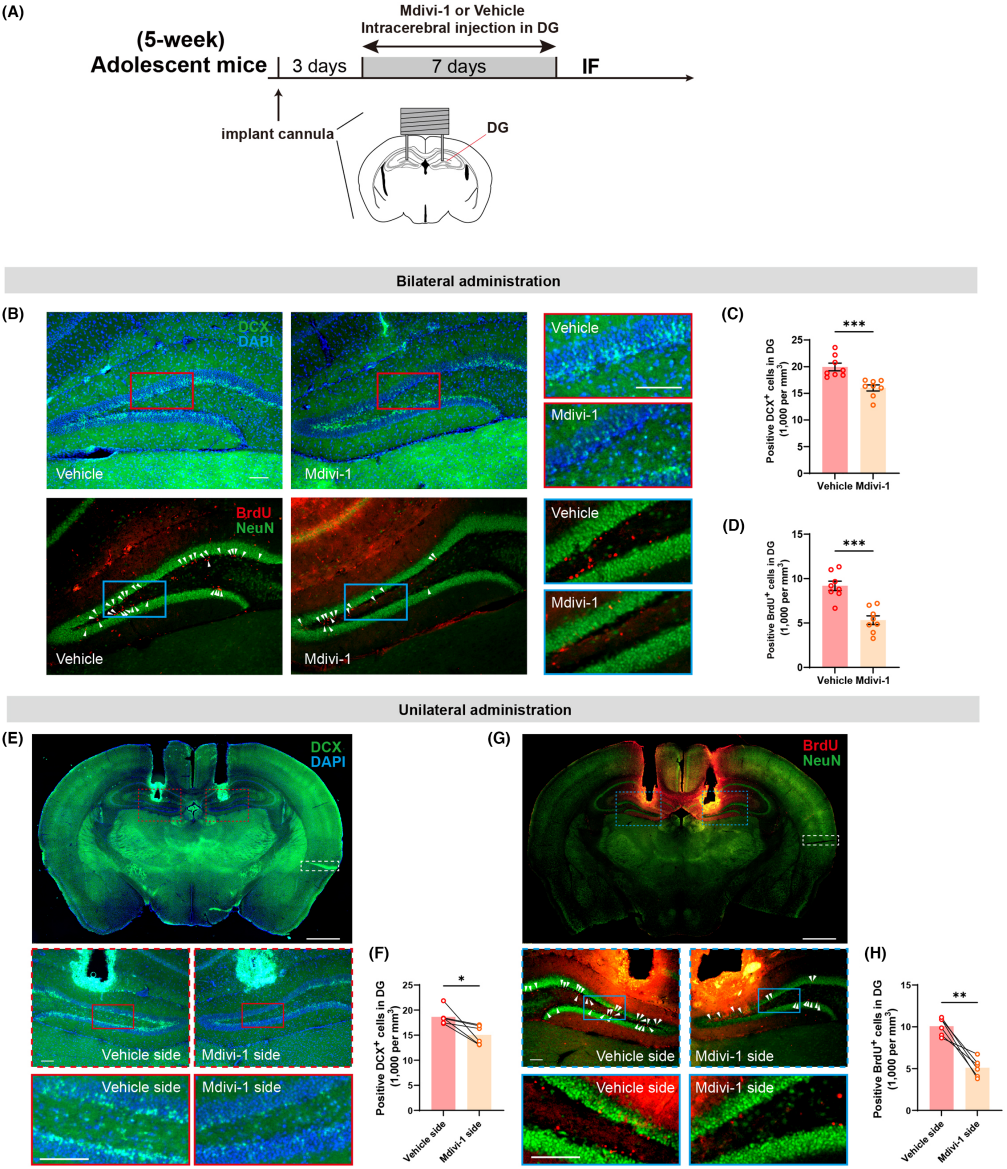
Effects of Mdivi‐1 on neurogenesis in adolescent mice. (A) Experimental diagram of intracerebral injection of Mdivi‐1 or ACSF. (B) Representative images of DCX, BrdU, and NeuN expression in the hippocampal DG of adolescent mice after bilateral administration of Mdivi‐1 or ACSF; scale bar (both in rimless and red/blue edge images), 100 μm. (C, D) Quantification from DCX and BrdU staining revealed the bilateral administration of Mdivi‐1 reduced neurogenesis in the hippocampal DG (*n* = 8 mice/group; for DCX, unpaired *t*‐test, *t*_(14)_ = 4.292, *p = 0.0007; for BrdU, unpaired *t*‐test, *t*_(14)_ = 5.363, *p = 0.0001). (E, G) Illustrative images depicting the expression of DCX, BrdU, and NeuN in the hippocampal DG of adolescent mice subsequent to the unilateral microinjection of Mdivi‐1; dotted box, the marker of the Mdivi‐1 side; scale bar, 1 mm (top) and 100 μm (middle and bottom). (F, H) Quantitative analysis from DCX and BrdU staining showed decreased newborn neurons within the unilateral DG where Mdivi‐1 was injected (*n* = 6 mice/group; for DCX, Wilcoxon test, *p = 0.0313; for BrdU, paired *t*‐test, *t*_(5)_ = 6.457, **p = 0.0013).

From the preceding research, it can be concluded that UMI‐77, as an inducer of mitophagy, may enhance spatial memory in adult mice by upregulating neurogenesis. To further substantiate the critical role of mitophagy in neurogenesis and spatial memory, intracerebral injections of the mitophagy inhibitor Mdivi‐1 were administrated into the DG of adolescent mice to evaluate changes in neurogenesis and spatial memory. At 5 weeks of age, mice underwent cannula implantation into the DG, with drug administration commencing 3 days later (Figure 6A). Initial bilateral microinjections of Mdivi‐1 or artificial cerebrospinal fluid (ACSF) in adolescent mice revealed significant inhibition of neurogenesis following 7 days of Mdivi‐1 treatment (Figure 6B–D). To more clearly delineate the impact of Mdivi‐1 on the number of newborn neurons, unilateral microinjections of Mdivi‐1 were performed, using ACSF injections as controls on the contralateral side. The results indicated a significant inhibition of neurogenesis in the DG following Mdivi‐1 administration (Figure 6E–H), establishing a correlation between neurogenesis and the level of mitophagy.

**FIGURE 7:**
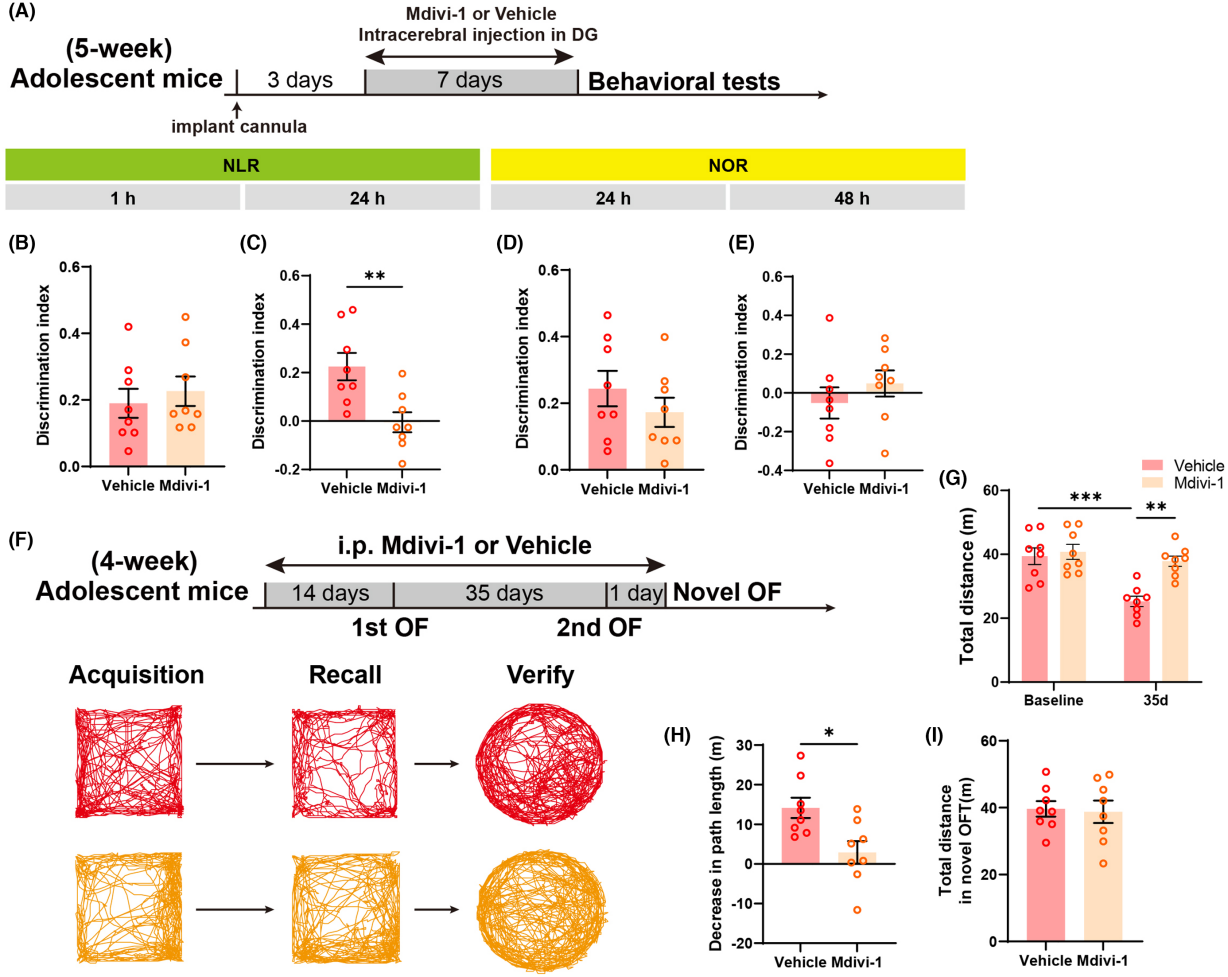
Effect of Mdivi‐1 on spatial memory in adolescent mice. (A) Experimental diagram of cognitive‐related behavioral tests when intracerebral injecting drug. (B, C) The Mdivi‐1 group showed a significant decrease in discrimination index of NLR at 24 h after training compared to the vehicle group, but not at 1 h post‐training (*n* = 8 mice/group; for 24 h, unpaired *t*‐test, *t*_(14)_ = 3.276, *p = 0.0055). (D, E) There was no significant difference in the discrimination index of NOR at 24 h and 48 h after training between adolescent mice intracerebral injected with Mdivi‐1 and vehicle (*n* = 8 mice/group). (F) Experimental diagram (top) and example exploration traces of NAPR (bottom) when using Mdivi‐1 or vehicle. (G) In the NAPR experiment, the vehicle‐treated adolescent mice exhibited a significant decrease in locomotor activity upon their second entry into the open field compared to their initial entry, as well as when compared to the second entry of the Mdivi‐1‐treated mice; but the Mdivi‐1‐treated adolescent mice showed no significant difference between two successive tests (*n* = 8 mice/group; two‐way ANOVA followed by Bonferroni's post hoc test, interaction of two factors: *F*_(1,28)_ = 7.186, *p* = 0.0122; for two groups after treatment of vehicle, baseline vs. 35 days, ***p = 0.0003; for two groups tested after 35 days, vehicle vs. Mdivi‐1, **p = 0.0013). (H) The average change (Δ) of total distance between the first two visits showed a significant difference between treatments of Mdivi‐1 and vehicle to adolescent mice (*n* = 8 mice/group; unpaired *t*‐test, *t*_(14)_ = 2.933, *p = 0.0109). (I) In the third visit, the novel open field, there was no significant difference in the total distance traveled between subjects that received treatment with UMI‐77 and vehicle (*n* = 8 mice/group).

Consistent with previous findings utilizing a mitophagy inducer, the application of Mdivi‐1 (Figure 7A) to inhibit neurogenesis in the DG during adolescence resulted in a significant decline in spatial recognition ability in 24 h NLR (Figure 7C), without affecting spatial recognition in 1 h NLR (Figure 7B) or object recognition ability in the 24 h and 48 h NOR (Figure 7D,E). Furthermore, Mdivi‐1 did not impact the performance of adolescent mice in the open field and Y‐maze tests (Figure S2). In the NAPR, mice received intraperitoneal injections of Mdivi‐1 or saline in advance for 2 weeks and continued maintenance (Figure 7F). On the 35th day, upon re‐entering the familiar arena, the control group exhibited a significant decrease in total distance compared to the initial entry, a phenomenon not observed in the Mdivi‐1 group. Additionally, during the second entry (35 days), the Mdivi‐1 group showed a significant increase in total distance compared to the control group (Figure 7G). There was also a significant difference in the decrease in path length between the Mdivi‐1 and vehicle groups (Figure 7H). In the subsequent day's novel open field test, the spontaneous activity of the Mdivi‐1 group was comparable to those of the vehicle group (Figure 7I). These findings collectively suggest that downregulating mitophagy in the DG through Mdivi‐1 impacts neurogenesis in adolescent mice, subsequently influencing the maintenance of spatial memory.

### 3.7 Mitophagy regulates neurogenesis and spatial memory independent of glial cell activation

Previous studies suggested that glial cells in the hippocampus played a crucial role in regulating spatial memory.^25^, ^29^, ^30^, ^31^ Interestingly, the function of glial cells is also implicated in neurogenesis.^32^, ^33^, ^34^ Therefore, we aimed to investigate whether alterations in mitophagy affect the production of newborn neurons via regulating glial cell activity. Immunofluorescence assays indicated that the quantity of glial cells is comparable between adolescent and adult mice (Figure S3A,B). Furthermore, the administration of the mitophagy inducer UMI‐77 and the inhibitor Mdivi‐1, at corresponding doses used in the aforementioned study, does not impact the number of glial cells. Consequently, the regulation of neurogenesis and spatial memory by mitophagy appears to be independent of the activity of glia cells (Figure 3C–F).

## 4 DISCUSSION

**FIGURE 8:**
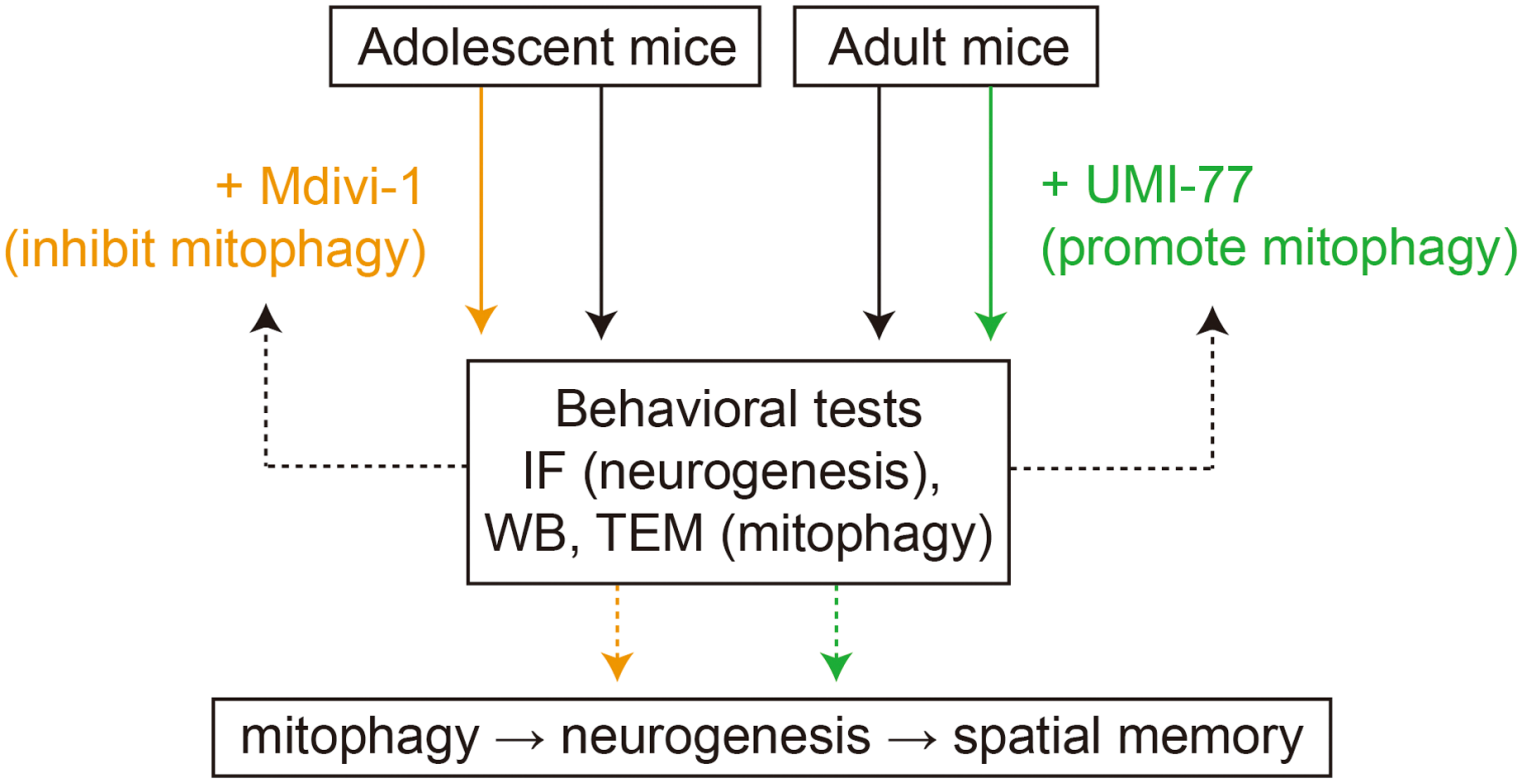
The flowchart of the experimental procedures.

The mechanisms influencing neurogenesis during developmental phases, as well as the behavioral implications of such alterations, remain unclear. We conducted our study following a detailed experimental protocol, as illustrated in Figure 8, to investigate these dynamics. We initially documented differences in spatial memory between adolescent and adult mice using the NLR and NAPR tests. Observations indicated a decline in neurogenesis and corresponding mitophagy in the hippocampus of adult mice compared to adolescent subjects. Treatment with UMI‐77, a mitophagy inducer, in adult mice led to an increase in mitophagy, enhanced neurogenesis, and extended spatial memory duration. Conversely, administering Mdivi‐1, a mitophagy inhibitor, to adolescent mice resulted in decreased hippocampal neurogenesis and shortened spatial memory retention. These findings suggested that the alteration of hippocampal mitophagy throughout the developmental period played a critical role in regulating neurogenesis and influencing the duration of spatial memory.

The behavioral differences in the developmental process of mice have been reported, with a considerable focus on cognitive function. Both juvenile and adolescent mice exhibited better cognitive flexibility compared to adult mice,^35^ while adolescent mice also demonstrated increased risk‐taking behavior and social preference.^36^ However, their working memory and temporal order object recognition abilities declined compared to adult mice.^37^ In the present study, we found that adolescent mice exhibit longer spatial memory duration. Although recent reports have suggested that spatial memory in juvenile mice is imprecise, by adolescence, spatial memory accuracy has essentially reached adult levels,^38^ displaying an advantage in spatial memory duration over adulthood from our results. No difference in spatial memory recognition was noted in the previous related report,^37^ possibly because a single time interval in NLRs may not be sufficient to detect diversity in spatial memory duration. Additionally, our other results revealed that there were no significant differences in object recognition ability, working memory, locomotor activity, or anxiety‐like behaviors between adolescent and adult mice, but these conclusions cannot be definitive due to the relatively limited scope of relevant behavioral tests.

In the current research, we reported enhanced spatial memory persistence in adolescent mice compared to adult mice. Spatial memory is closely associated with the hippocampal region,^39^ and studies have shown that impaired adult neurogenesis can lead to spatial memory impairments in various animal disease models.^21^, ^40^, ^41^ Consistent with observations in postmortem human brain,^42^ the capacity for hippocampal neurogenesis in mice diminishes significantly from adolescence to adulthood,^43^ a phenomenon also corroborated in our study, as evidenced by a marked reduction in DCX^+^ and BrdU^+^ cells in the DG of adult mice. We also examined the role of mitochondrial dynamics in neurogenesis during development. Mitochondrial dynamics, constituting essential mechanisms by which the organism safeguards mitochondrial function, serve as crucial regulatory factors in adult neurogenesis under pathological conditions.^20^, ^21^, ^22^, ^23^, ^24^ However, their role as a modulator of neurogenesis during developmental processes remains unclear. Our results indicate that there is a decline in mitophagy from adolescence to adulthood, along with a simultaneous decrease in mitochondrial fusion. Furthermore, we found that the decline in mitophagy primarily involves a reduction in ubiquitin‐dependent mitophagy, rather than receptor dependent. Given that impaired mitochondrial dynamics can contribute to adult neurogenesis impairments under pathological conditions, and our results suggest that adolescent mice have a more robust system for maintaining mitochondrial function compared to adult mice, we propose that this safeguard system may be crucial for supporting high levels of neurogenesis during adolescence.

We verified our hypothesis by modulating mitochondrial dynamics in vivo. Selective pharmacological agents directly regulating mitophagy are only a few, one of which is UMI‐77. It acts on the MCL‐1 receptor of mitochondria to promote mitophagy and effectively ameliorate Alzheimer's disease phenotypes and renal fibrosis.^28^, ^44^ In our study, systemic administration of UMI‐77 upregulated mitophagy in the hippocampus of adult mice. Interestingly, this also led to a rise in the level of adult neurogenesis. Previous research has demonstrated that such biological changes enhanced cognitive functions like spatial memory in animals with various pathological conditions.^21^, ^23^, ^40^ However, it remains unclear whether the upregulation of neurogenesis under normal physiological conditions affects hippocampus‐related cognitive functions in adult mice. Our behavioral results demonstrated that UMI‐77 increased the duration of spatial memory in adult mice. These findings suggest that enhancing mitophagy promotes neurogenesis in the adult hippocampus and extends spatial memory duration. In contrast, Mdivi‐1, a selective DRP1 inhibitor, inhibits mitochondrial fission and mitophagy and can cross the blood–brain barrier.^45^ Our results showed that intracerebral injection of Mdivi‐1 significantly inhibited hippocampal neurogenesis in adolescent mice and reduced the duration of spatial memory in NLR and NAPR tests. These findings indicate that inhibiting mitophagy decreased hippocampal neurogenesis in adolescent mice and reduced spatial memory duration. To demonstrate the specificity of the hippocampal DG in our study, we chose intracerebral injection of Mdivi‐1 in this part of the experiment to avoid the indirect effects of changes in mitophagy in other brain regions on hippocampal neurogenesis and spatial memory. However, considering the interaction between instability of implanted cannula and the excessively long experimental dosing period (>35 days), intraperitoneal administration was used in the NAPR tests. Hence, we conclude that during the development from adolescence to adulthood in mice, hippocampal mitophagy regulates neurogenesis to modulate the maintenance capacity of spatial memory. In our previous studies, we also observed a decrease in mitochondrial fusion in adulthood. However, due to the current lack of the regulatory drugs that can clearly cross blood–brain barrier, we did not validate the role of mitochondrial fusion and cannot exclude its involvement in neurogenesis during development.

Memory can be categorized into long‐term memory and short‐term memory based on how long information is retention.^46^ In mice, long‐term memory lasts for several weeks or more, whereas short‐term memory only lasts for a few days. The transition between long‐term and short‐term memory is believed to involve continuous communication between the hippocampus and the cortex.^47^ Our results from NLR and NAPR tests indicate that mitophagy‐mediated neurogenesis in the DG is crucial for maintaining both short‐term and long‐term memory, supporting the neural networks underlying memory. This finding aligns with previous research demonstrating the involvement of the hippocampal DG in the duration transition of memory.^48^ Adolescent neurogenesis, as well as enhanced adult neurogenesis following pharmacological intervention, may contribute to the formation of additional memory engrams or increase the duration of their maintenance, thereby prolonging the retention of spatial memory. The process of memory encompasses encoding, consolidation, retrieval, and forgetting,^49^ with the hippocampal DG playing a vital role in these processes. It has been reported that neurogenesis‐mediated synaptic remodeling promotes memory forgetting,^50^, ^51^ which reduces the duration of memory retention and seems to contradict our conclusion. In fact, given our dosing period, the regulatory effect of mitochondrial dynamics on neurogenesis may primarily occur during the encoding and consolidation stages of memory. Since our intervention was not implemented after memory acquisition, the results cannot assess the level of memory forgetting.

As the behavioral approaches of this research are limited, it cannot be ruled out that changes in hippocampal neurogenesis mediated by mitophagy may contribute to the behavioral differences reported in other studies between adolescent and adult mice.^36^, ^37^, ^52^, ^53^ Furthermore, our current work cannot definitively determine whether mitochondrial dynamics within neural stem cells directly influence neurogenesis or within neighboring cells play an indirect role. In the hippocampus, The functions performed by microglia and astrocytes in the hippocampus are crucial for the regulation of neurogenesis,^32^, ^33^, ^34^ while their roles are also associated with spatial memory processing such as acquisition and forgetting.^25^, ^29^, ^30^, ^31^ Although our work did not reveal significant alterations in the quantity of astrocytes and microglia through immunofluorescence, this is insufficient to completely rule out their involvement in mitochondrial regulation of neurogenesis. The specific mechanisms by which mitochondrial dynamics in the DG regulate neurogenesis require further elucidation.

But to be sure, current research has shed further light on the relationship between mitophagy and hippocampal neurogenesis, offering new therapeutic targets for diseases associated with adult neurogenesis impairment. Additionally, the aforementioned results also suggested that UMI‐77 may hold promise as a lead compound for treating these diseases from a mitochondrial dynamics perspective.

## AUTHOR CONTRIBUTIONS

S.H., H.X., and L.X. were responsible for the overall experimental design. X.M., L.X., A.T., and Y.T. performed the behavioral tests. X.C., Y.S., X.Z., and X.M. performed the immunofluorescence staining, western blotting, and TEM. D.Z. microinjected the drug for intervention. H.Z., J.L., S.S., and Y.S. performed the data analysis. L.X., X.C., and S.S. outlined and wrote the manuscript, which was reviewed by all authors.

## CONFLICT OF INTEREST STATEMENT

All authors declare that they have no conflicts of interest.

## Supporting information


Figures S1–S4.

Table S1.

## Data Availability

All relevant data supporting the key findings of this study are available within the article and its Supplementary Information files.
