# Supplementary material for: Hippocampal mitophagy contributes to spatial memory via maintaining neurogenesis during the development of mice
Source: CNS Neurosci Ther. 2024 Jun 17;30(6):e14800. doi: 10.1111/cns.14800 (PMC11183181; doi:10.1111/cns.14800)

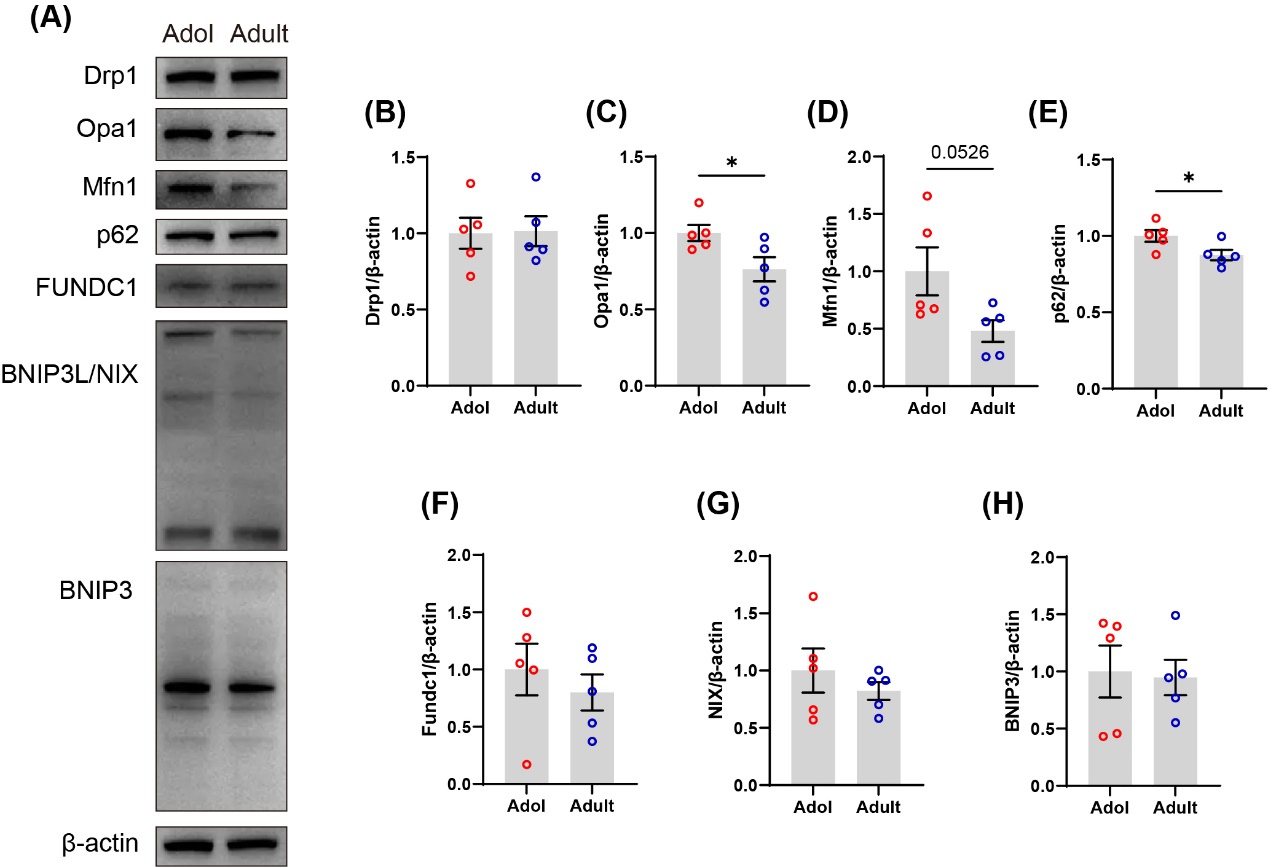


**FIGURE S1 Differences in protein expression levels of mitochondrial fusion, fission, and mitophagy in the hippocampus of adolescent and adult C57 mice.**

(A) Representative western blots of mitochondria/mitophagy-related proteins in the hippocampus between adolescence and adulthood of male C57 mice.

(B) There were no significant differences observed in the expression of the mitochondrial fission protein DRP1 between adolescent and adult C57 mice (n = 5 mice/group).

(C,D) Quantitative results indicate a lower expression level of the mitochondrial fusion proteins Opa1 and Mfn1 in the hippocampal region of adult mice compared to adolescent mice (n = 5 mice/group; for Opa1, unpaired t-test, t_(8)_=2.476, *p=0.0383; for Mfn1, unpaired t-test, t_(8)_=2.273, p=0.0526).

(E) Compared to adolescent mice, the expression of the autophagy protein P62 significantly decreases in the hippocampus of adult mice (n = 5 mice/group; unpaired t-test, t_(8)_=2.431, p=0.0411).

(F-H) No significant difference was observed in the expression levels of the mitophagy receptor proteins Fundc1, NIX, and BNIP3 within the hippocampus when comparing adolescent and adult C57 mice (n = 5 mice/group).


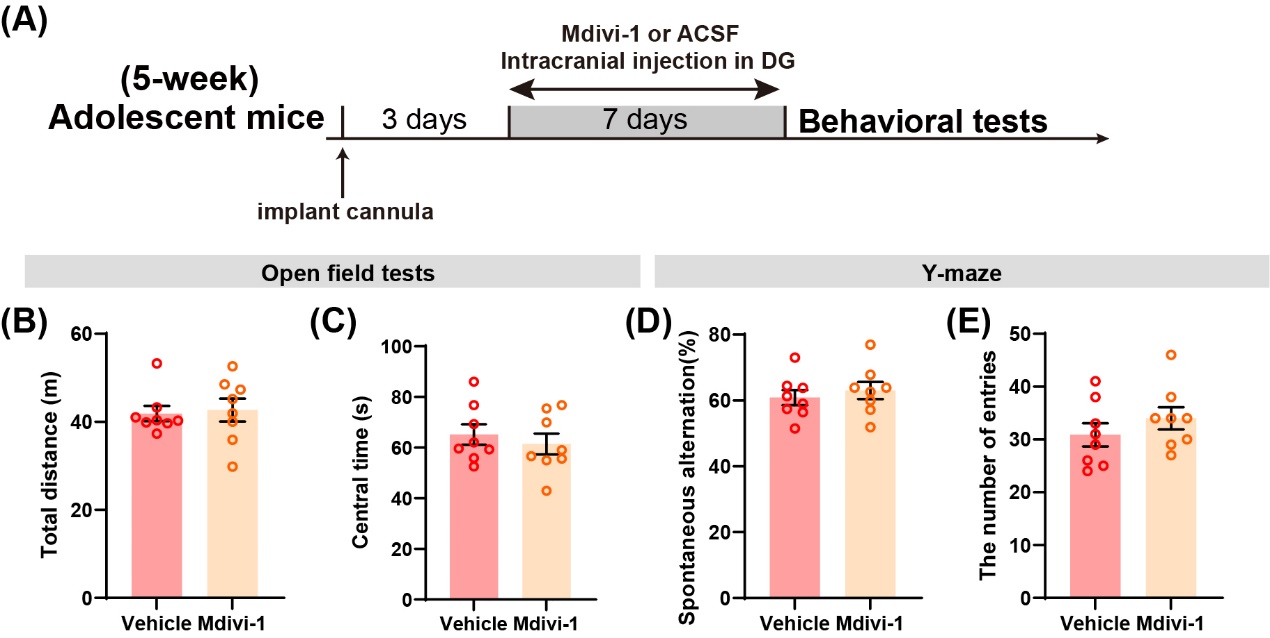


**FIGURE S2 Effects of Mdivi-1 on the performance of OFT and Y-maze in adolescent mice.**

(A) Experimental diagram of cognitive-related behavioral tests when intracerebral injecting drug.

(B,C) The total distance and central time in OFT showed no difference between treatments of Mdivi-1 and vehicle to adolescent mice (n = 8 mice/group).

(D,E) There were no significant difference observed in the performance of Y-maze between the adolescent mice treated with Mdivi-1 and vehicle (n = 8 mice/group).


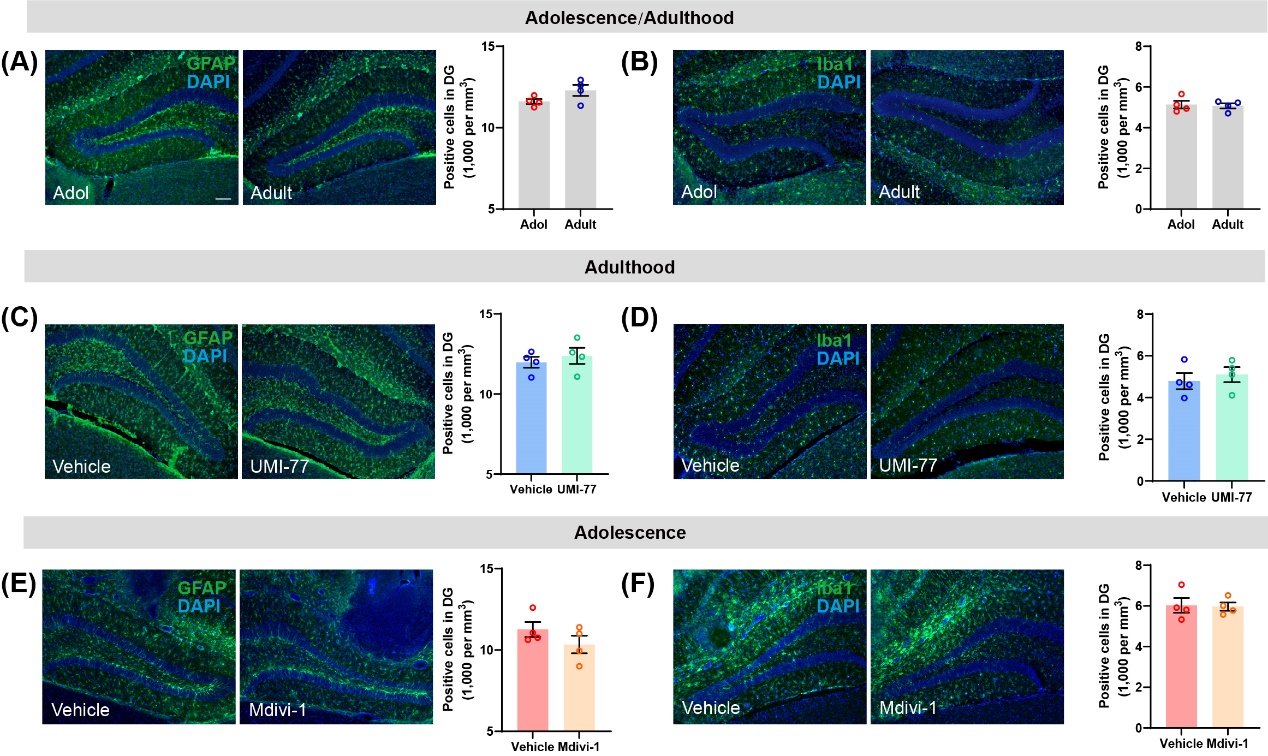


**FIGURE S3 The expression of glial cells during the process of adulthood or after the application of UMI-77 and Mdivi-1.**

(A,B) The quantitative results of immunofluorescence showed no difference in the number of astrocytes and microglia between adolescence and adulthood in mice (n = 4 mice/group).

(C,D) Quantification from immunofluorescence staining displayed the expression levels of astrocyte or microglia in hippocampal DG were not affected by UMI-77 (n = 4 mice/group).

(E,F) Quantitative analysis from DCX and BrdU staining showed the number of glial cells did not change significantly after microinjection of Mdivi-1 (n = 4 mice/group).

**FIGURE S4 Original western blotting images reported in this paper.**


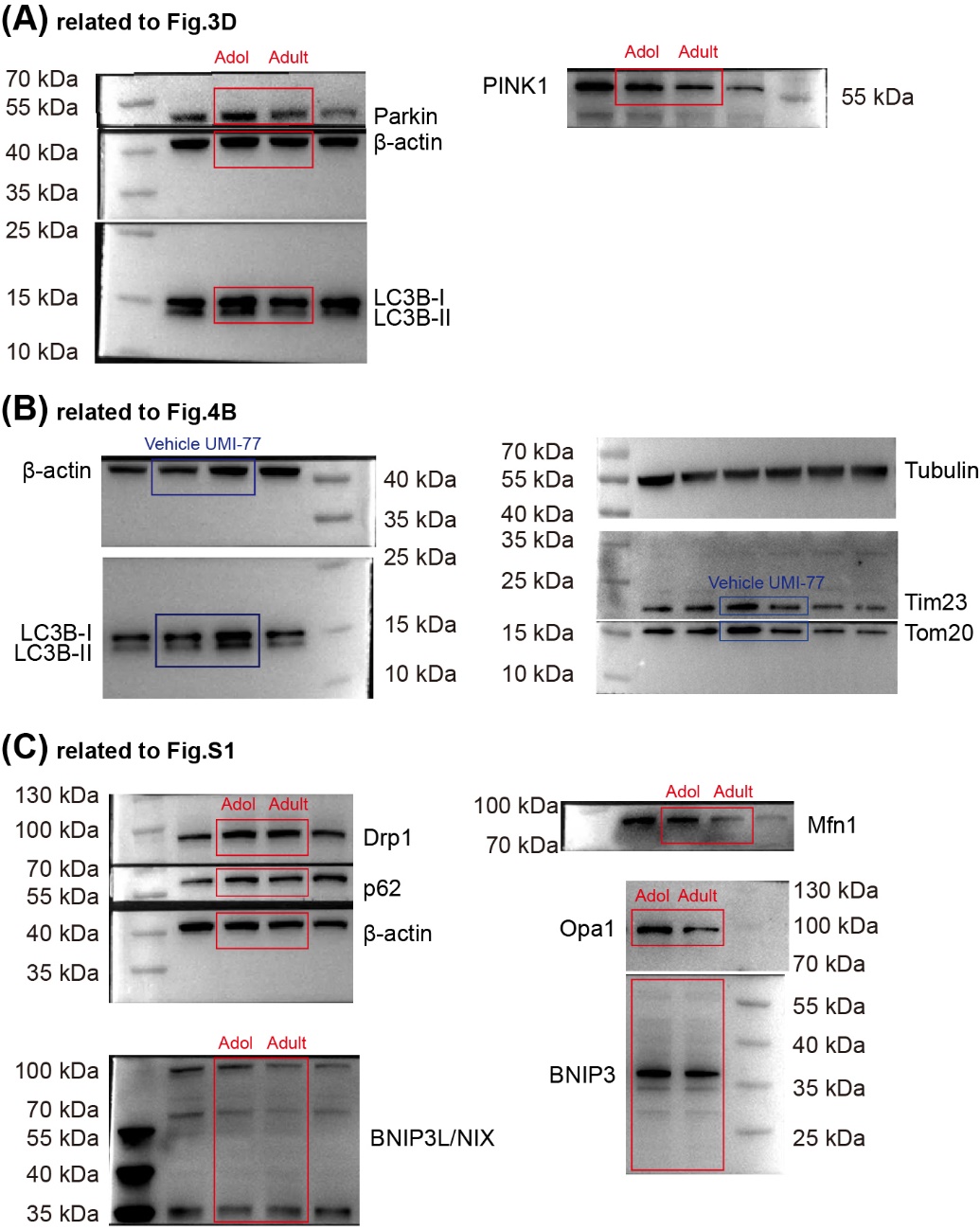

Supplement: Supplementary file 1 — Figures S1–S4. [file CNS-30-e14800-s002.docx]
